# 191. Long-term Utility of Clinical Nudges within Clostridioides difficile Diagnostic Stewardship

**DOI:** 10.1093/ofid/ofaf695.066

**Published:** 2026-01-11

**Authors:** Kenneth D Long, Molly E Fleece, Ryan B Ruhr, Megan Amerson-Brown, Sixto M Leal, Rachael A Lee

**Affiliations:** The University of Alabama at Birmingham, Birmingham, Alabama; University of Alabama at Birmingham, Birmingham, Alabama; University of Alabama at Birmingham, Birmingham, Alabama; University of Alabama in Birmingham Heersink School of Medicine, Birmingham, Alabama; University of Alabama at Birmingham, Birmingham, Alabama; University of Alabama at Birmingham, Birmingham, Alabama

## Abstract

**Background:**

IDSA recommends that, prior to testing for *Clostridiodes difficile* in patients with new-onset diarrhea, clinicians should consider other potential causes, such as recent laxative use. Clinical decision support systems (CDSS) can nudge providers to reduce inappropriate CDI testing and 2-step testing algorithms can optimize diagnostic accuracy. We assessed the long-term clinical utility of clinical nudges for CDI diagnostic stewardship.

**Figure:**
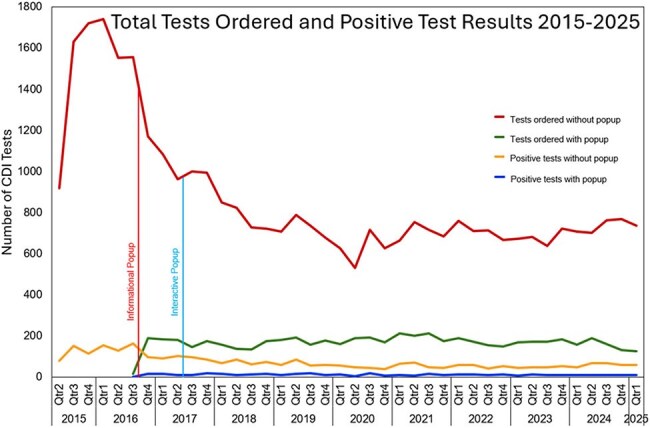

**Figure:**
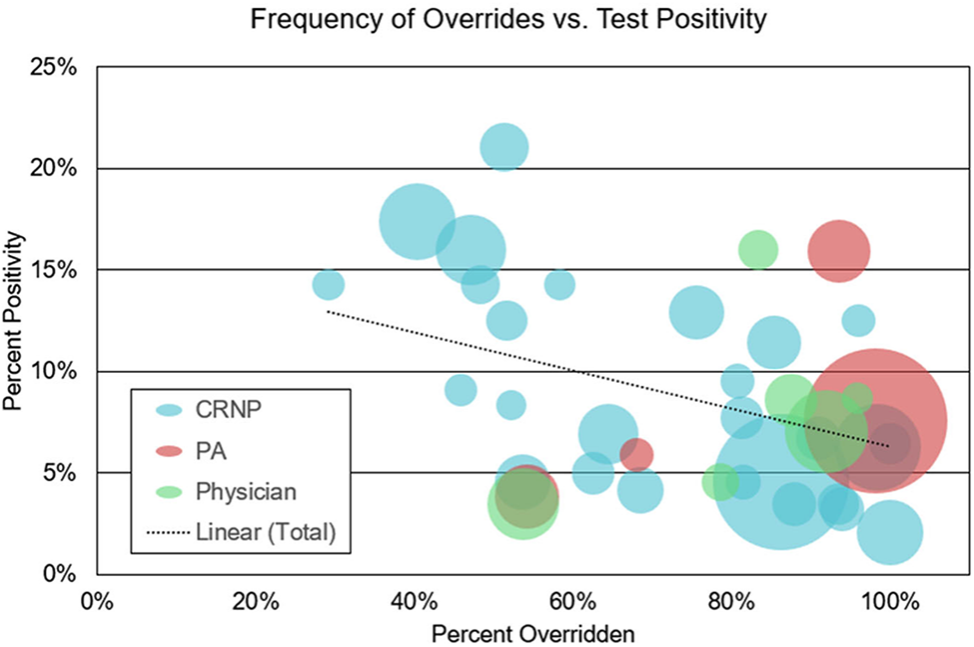

**Methods:**

A retrospective analysis of inpatient CDI tests (n=44,677) ordered for 10 years after the introduction of 2-step diagnostic testing (toxin→DNA-NAAT) in the 2^nd^ quarter of 2015 (Q2 2015) to evaluate the long-term impact of a CDSS nudge for providers to reconsider testing in patients receiving laxatives in the 48 hours prior. Univariate and multivariate analyses were performed via Pearson’s chi-squared test.

**Results:**

An informational popup (Q3 2016) produced a marked decrease in CDI testing (Fig. 1). It was transitioned to an interactive popup prompting either cancelation or acknowledgment that a “positive result will be of no value” (Q2 2017). The test positivity rate was significantly lower (7.0% vs. 8.5%, P < .001) for inpatients who triggered the popup (n=9231) versus those who did not during the same period.

No significant change in overall propensity to override the CDSS popup was observed over the study period (µ=57%). Popups were most frequently overridden in ICUs (62.0%), compared with Floor and Post-Acute Units, at 56.1 and 58.0%, respectively (p< .0001). There was a significant difference in rate of override between ordering provider types (APP vs. Attending vs. Resident) with rates of 59.4%, 57.2% and 55.5%, respectively (p< .01). In looking at individual providers, 2.5% of providers (n=50) were responsible for 20% of popup triggers, with a negative correlation between frequency of override and ordered test positivity (Fig. 2).

**Conclusion:**

While it has been shown that CDSS nudges can decrease diagnostic ordering, here we show that implementation of a CDSS yielded a statistically significant decrease in test positivity for a patient population at low-risk for CDI. Subgroup analyses looking at provider, unit, and level of care provide opportunities for targeted educational intervention to further improve diagnostic stewardship.

**Disclosures:**

All Authors: No reported disclosures

